# Diversity and distribution of biosynthetic gene clusters in agricultural soil microbiomes

**DOI:** 10.1128/msystems.01263-23

**Published:** 2024-03-12

**Authors:** Zhiguo Zhang, Lu Zhang, Lihan Zhang, Haiyan Chu, Jizhong Zhou, Feng Ju

**Affiliations:** 1 College of Environmental and Resource Sciences, Zhejiang University, Hangzhou, Zhejiang, China; 2 Key Laboratory of Coastal Environment and Resources Research of Zhejiang Province, School of Engineering, Westlake University, Hangzhou, Zhejiang, China; 3 Center of Synthetic Biology and Integrated Bioengineering, Westlake University, Hangzhou, Zhejiang, China; 4 Institute of Advanced Technology, Westlake Institute for Advanced Study, Hangzhou, Zhejiang, China; 5 Institute of Natural Sciences, Westlake Institute for Advanced Study, Hangzhou, Zhejiang, China; 6 Department of Chemistry, Key Laboratory of Precise Synthesis of Functional Molecules of Zhejiang Province, School of Science, Westlake University, Hangzhou, Zhejiang, China; 7 State Key Laboratory of Soil and Sustainable Agriculture, Institute of Soil Science, Chinese Academy of Sciences, Nanjing, China; 8 Department of Microbiology and Plant Biology, Institute for Environmental Genomics, University of Oklahoma, Norman, Oklahoma, USA; 9 School of Life Sciences, Westlake University, Hangzhou, Zhejiang, China; Leiden University, Leiden, the Netherlands

**Keywords:** agricultural soil microbiome, biosynthetic gene clusters, diversity, Secondary metabolite, Microbial community assembly

## Abstract

Bacterial secondary metabolites serve as an important source of molecules for drug discovery. They also play an important function in mediating the interactions of microbial producers with their living environment and surrounding organisms. However, little is known about the genetic novelty, distribution, and community-level impacts of soil bacterial biosynthetic potential on a large geographic scale. Here, we constructed the first catalog of 11,149 biosynthetic gene clusters (BGCs) from agricultural soils across China and unearthed hidden biosynthetic potential for new natural product discovery from the not-yet-cultivated soil bacteria. Notably, we revealed soil pH as the strongest environmental driver of BGC biogeography and predicted that soil acidification and global climate change could damage the biosynthetic potential of the soil microbiome. The co-occurrence network of bacterial genomes revealed two BGC-rich species, i.e., *Nocardia niigatensis* from Actinobacteriota and *PSRF01* from Acidobacteriota, as the module hub and connector, respectively, indicating their keystone positions in the soil microbial communities. We also uncovered a dominant role of BGC-inferred biotic interactions over environmental drivers in structuring the soil microbiome. Overall, this study achieved novel insights into the BGC landscape in agricultural soils of China, substantially expanding our understanding of the diversity and novelty of bacterial secondary metabolism and the potential role of secondary metabolites in microbiota assembly.

**IMPORTANCE:**

Bacterial secondary metabolites not only serve as the foundation for numerous therapeutics (e.g., antibiotics and anticancer drugs), but they also play critical ecological roles in mediating microbial interactions (e.g., competition and communication). However, our knowledge of bacterial secondary metabolism is limited to only a small fraction of cultured strains, thus restricting our comprehensive understanding of their diversity, novelty, and potential ecological roles in soil ecosystems. Here, we used culture-independent metagenomics to explore biosynthetic potentials in agricultural soils of China. Our analyses revealed a high degree of genetic diversity and novelty within biosynthetic gene clusters in agricultural soil environments, offering valuable insights for biochemists seeking to synthesize novel bioactive products. Furthermore, we uncovered the pivotal role of BGC-rich species in microbial communities and the significant relationship between BGC richness and microbial phylogenetic turnover. This information emphasizes the importance of biosynthetic potential in the assembly of microbial communities.

## INTRODUCTION

Many microorganisms encode biosynthetic gene clusters (BGCs) to produce diverse secondary metabolites (also called specialized metabolites), such as antibiotics, quorum-sensing molecules, and siderophores (1, 2). These biomolecules not only have versatile applications in modern medicine and biotechnology (3, 4) but also play an important ecological role in mediating the interactions of microbial producers with their living environment and sympatric organisms (4–6). However, due to the vast majority of environmental microbes remaining uncultivated (7), our knowledge of secondary metabolism in soil bacteria is limited to only a small fraction of cultured strains, thereby restricting our complete understanding of the biotechnological relevance and ecological importance of secondary metabolites in soil microbiomes and ecosystems (8).

Metagenomics, with technological advances over the past decade, has offered new culture-independent paths to explore the biosynthetic potential of environmental microbiomes comprehensively. Recent culture-independent metagenomic studies have revealed the extensive presence of BGC-rich taxa and novel BGCs in various soil ecosystems, including soils from grasslands (9, 10), Antarctic (11), forests (10, 12), and biological crust (13). Furthermore, they revealed that vegetation type, soil Horizons, and nutrient availability could shift the biosynthetic profiles of soil microbiome (10, 12, 14). These results substantially expand our knowledge of the genetic diversity and driving factors of soil BGCs. However, agricultural soils, which are subject to consecutive anthropogenic disturbances such as chemical fertilizer, pollution, and tillage, have not been explored with regard to the diversity and novelty of their secondary metabolic potential. In addition, how global BGC profiles respond to soil physiochemical properties, climatic characteristics, and geographic variables at a continental scale remains unknown. Some previous amplicon-based studies have suggested the roles of ecological and evolutionary pressures in driving the distribution of nonribosomal peptide synthetase (NRPS) and polyketide synthase (PKS) (15–18). However, amplicon-based BGC profiling is mainly restricted to the NRPS and PKS, which have conserved domains, while terpenes and ribosomally synthesized and post-translationally modified peptides (RiPPs) are difficult to capture using domain-specific and primer-based PCR approaches. In contrast, metagenomics can recover the full genetic context of BGCs belonging to different classes in soil microbiomes.

In addition to the environmental forces driving BGC distribution, their impacts on shaping environmental microbial communities are still not clear. Previous studies of multiple bacterial strains suggested that secondary metabolites synthesized by microbial BGCs could drive interspecies interaction networks (19). Moreover, antagonistic interactions mediated by secondary metabolites were also observed among a diverse range of bacterial lineages (20–23). To date, little is known about the *in situ* outcomes of secondary metabolomes synthesized by BGCs on environmental microbiota, although this knowledge is crucial for understanding and predicting microbial behaviors during community assembly and succession.

In this study, we aim to uncover potentially diverse unexplored BGCs and undescribed BGC-rich taxa in agricultural soils and environmental forces driving the biogeographical distribution of BGCs. We also hypothesize that BGC profiles could manipulate the assembly of microbial communities. To achieve these goals, we integrated metagenomics and 16S metabarcoding to explore the novelty, biogeography, and microbial community-level impacts of soil BGCs in agricultural land covering all climate zones of mainland China.

## RESULTS

### Agricultural soil microbiome encoded biosynthetic potential with high genetic diversity and novelty

**Fig 1:**
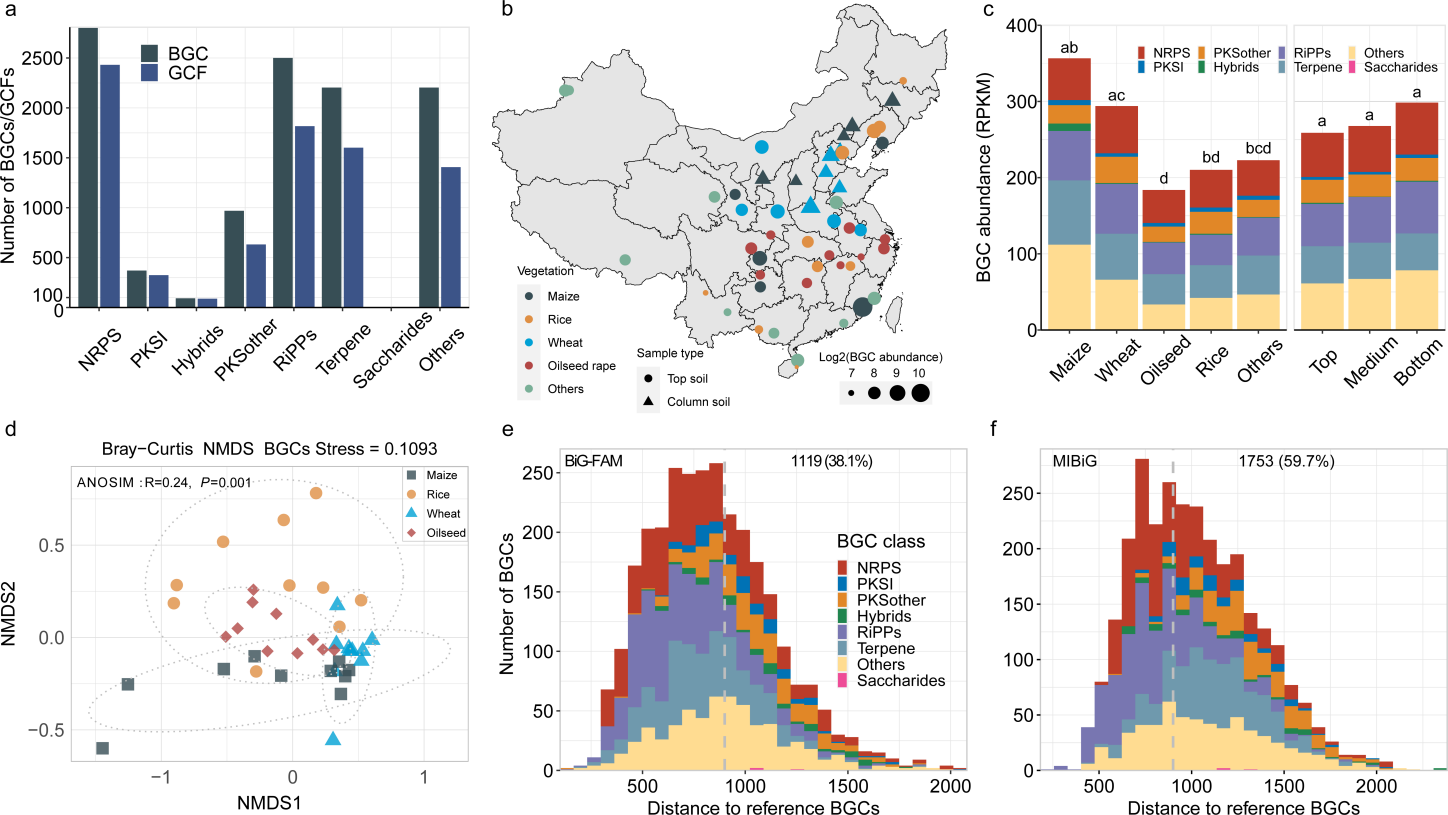
The biosynthetic gene clusters (BGCs) recovered from China agricultural soil metagenomes. (**a**) The number of BGCs and gene cluster families (GCFs) recovered from the soil metagenomes. (**b**) The relative abundance of BGCs in different sampling sites. Only the BGC abundances of top soils were shown. The map was plotted using R. (**c**) The composition of BGCs of samples with varying vegetation types (maize, wheat, oilseed rape, rice, and others) and soil depths (0–15 cm, 15–30 cm and 30–45 cm). Each vegetation type or soil depth had 10 samples. (**d**) Nonmetric multidimensional scaling plots depict Bray-Curtis distances between vegetation types based on the relative abundance of BGCs in soil metagenomes. (**e**) The novelty of BGCs compared with the computationally predicted BiG-FAM database. (**F**) The novelty of BGCs as compared with the experimentally validated MIBiG database.

The agricultural soil microbiotas are exposed to a wide range of exogenous chemicals and microbes as environmental and biotic drivers of its genetic diversity (24). However, the biosynthetic potential of agricultural soil bacteria and their potential as a reservoir of new natural products remain largely underexplored. To construct the catalog of biosynthetic gene clusters (BGCs) of agricultural soil microbiomes and further depict their biogeography and ecology, the antibiotics and secondary metabolite analysis shell (antiSMASH (25) were used to annotate 2.2 million scaffolds assembled from 70 agricultural soil samples across China (Fig. S1; Data set S1). In total, 11,149 BGCs were identified and clustered into 8,303 gene cluster families (GCFs), including NRPS, PKS/NRPS hybrids, PKSI (type I PKS), PKSother (mainly type II PKS and type III PKS), RiPPs, Terpene, Saccharides, and Others (Fig. 1a).

Consistent with the broad range in genetic diversity of biosynthetic potential in agricultural soils (2,087 to 3,991 BGCs, Fig. S2), we found that the total abundance of soil BGCs (coverage, × /Gb) also varied greatly among sampling sites, ranging from 126 to 1,368, reflecting enormous differences in biosynthetic potential among soil microbiomes (Fig. 1b). On average, the maize soil microbiome showed the highest BGC abundance (356.6), which was significantly higher than that of oilseed rape (183.8, Mann‒Whitney U test, *P* = 0.01). Similarly, the BGC abundance of the wheat soil microbiome (293.9) was significantly higher than those of oilseed rape (*P* = 0.0004) and rice (210.2, *P* = 0.03) (Fig. 1c). However, the BGC abundance was not significantly different (Wilcoxon signed-rank test, *P* > 0.05) among the top (258.9, 0–15 cm), middle (267.8, 15–30 cm), and bottom (298.5, 30–45 cm) soil layers (Fig. 1c). Furthermore, BGC compositions differed significantly among different vegetation types (Fig. 1d) and between pairwise vegetation types (PERMANOVA test, *P* < 0.05) (Table S1), indicating that the significant variations in biosynthetic potentials are potentially driven by the types of agricultural soil vegetation.

To evaluate the BGC novelty, only 3,947 BGCs (35.4% of all identified BGCs) encoded on scaffolds with a length of ≥5 kb were selected in order to reduce BGC fragmentation, as done previously (26, 27). Then, these BGCs were clustered into 2,938 GCFs so as to mitigate redundancy (i.e., the same BGC can be encoded in several scaffolds). The results showed that 38.1% of 2,938 GCFs showed no overlap (*d* > 900) with BGCs in the computationally predicted database BiG-FAM (28) (Fig. 1e), and 59.7% were not represented in the experimentally validated database MIBiG (29) (Fig. 1f). Considering that the novelty of BGCs located on contig edge will be underestimated (11), the overall novelty of BGCs in this study should be much higher because 92.7% of these GCFs were located on the contig edge (Data set S2). Therefore, we inspected 211 complete GCFs and found that 59.2% of GCFs were novel when compared against the BiG-FAM database, comparable to that of Antarctic soil (59.6%) (11). To further explore their potential for new drug discovery, we compared the chain length factor (CLF) sequences of 17 type II PKS (T2PKS) of this study with those of experimentally validated T2PKS curated by a recent study (30). Both sequence similarity and the phylogenetic cladogram showed that the CLFs of all the T2PKS retrieved from the agricultural soils had low sequence similarity (ranging from 0.37 to 0.79) with the CLFs of known T2PKS (Fig. S3), indicating their potential to produce diverse new aromatic polyketides.

### Genome-resolved metagenomics revealed uncharted BGC-rich taxa in agricultural soils

**Fig 2:**
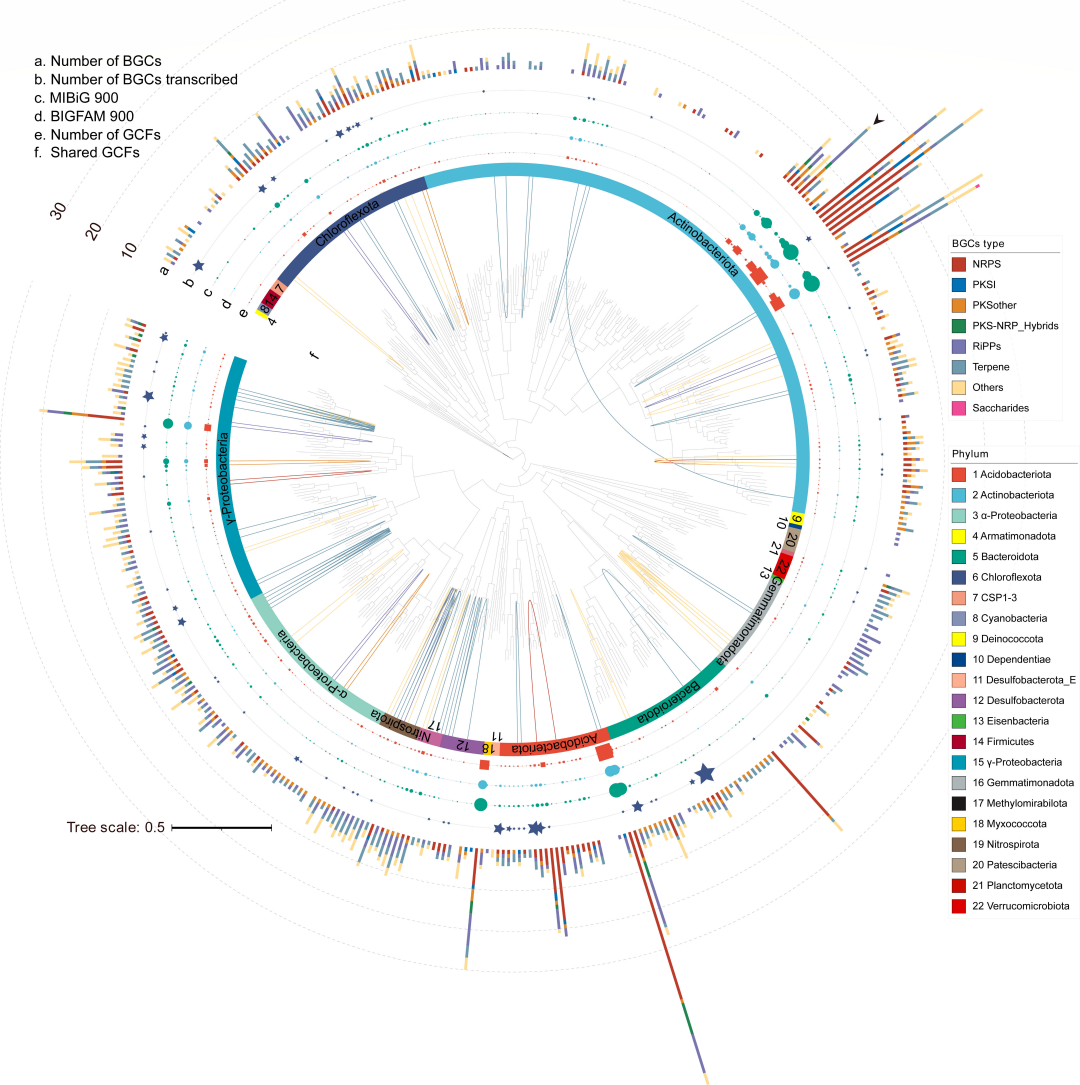
Phylogenetic distribution of 449 species-level representative bacterial MAGs. The phylogenetic tree was constructed based on 120 bacterial marker genes using GTDBTk and visualized in iTOL. Each colored ring indicates a taxonomic phyla group. The stacked bar shows the amounts of BGCs in each genome, and each color represents a BGC class. The square symbol indicates the amounts of GCFs in each genome. The green and blue circle symbols indicate the amounts of novel BGCs compared to the experimentally validated MIBiG database and the computationally predicted BiG-FAM database, respectively. The pentagon symbols indicate the number of BGCs transcribed in the nine metatranscriptomes. The arrow indicates the unclassified BGC-rich family. The inner solid lines indicate the GCFs shared by different bacterial genomes, and the lines are colored according to BGC types.

Having established that agricultural soil microbiomes harbor diverse BGCs with high novelty, we further placed the BGC diversity into their host genomic context, which is critical for predicting yet uncharacterized microbial lineages that encode new natural products. Previous genome-resolved metagenomic mining has revealed several underexplored BGC-rich taxa in grassland (9) and ocean ecosystems (27). To extensively resolve the taxonomy of BGC hosts, we constructed metagenome-assembled genomes (MAGs) using an optimized strategy that supplemented routine individual-sample with cross-sample *de novo* binning (see Methods), which substantially improved the total yields of MAGs by 30.4% (Fig. S1). Finally, a total of 510 eligible nonredundant MAGs were recovered as species-level representatives, including 61 archaeal MAGs (Fig. S4) and 449 bacterial MAGs (Fig. 2). The bacterial MAGs were broadly assigned to 20 phyla (Data set S3), such as Actinobacteriota (33.6%), γ-Proteobacteria (13.8%), and Acidobacteriota (6.2%). About 53.0% of these bacterial MAGs could be found in more than half of the 70 soil samples (Fig. S5), indicating their wide distribution in geographically and edaphically different soil environments. Among them, 386 bacterial MAGs encode 1,892 BGCs (Data set S4), such as NRPS (24.0%), terpene (22.5%), and RiPPs (20.1%).

To inspect the differences in biosynthetic potential between bacterial groups, we charted the phylogenetic relationships and BGC profiles of bacterial genomes (Fig. 2). The stacked histogram of the phylogenetic tree showed that bacterial groups are not equally rich in their biosynthetic potential (Fig. 2a). For example, Acidobacteriota encoded an average of 8.21 BGCs per genome, which was 2-fold higher than that of α-Proteobacteria (3.73) and γ-Proteobacteria (4.27). The composition of BGCs also varied greatly among the dominant bacterial phyla. NRPS accounted for 46.1% of the BGCs of Acidobacteriota, which was 2.6, 3.0, and 8.6 times higher than that of Bacteroidota, Chloroflexota, and α-Proteobacteria, respectively (Fig. S6). The results indicate substantial differences in the distribution of BGCs across taxa. This trait was also validated by previous findings that the majority of BGCs are unique to each phylum and that lower taxonomic ranks, such as species within a genus, are more likely to display uniform biosynthetic diversity than higher taxonomic ranks (31). This pattern was also supported by the sequence similarity network of BGCs, in which GCFs were mostly shared by genomes from the same bacterial genera (Fig. 2f).

**Fig 3:**
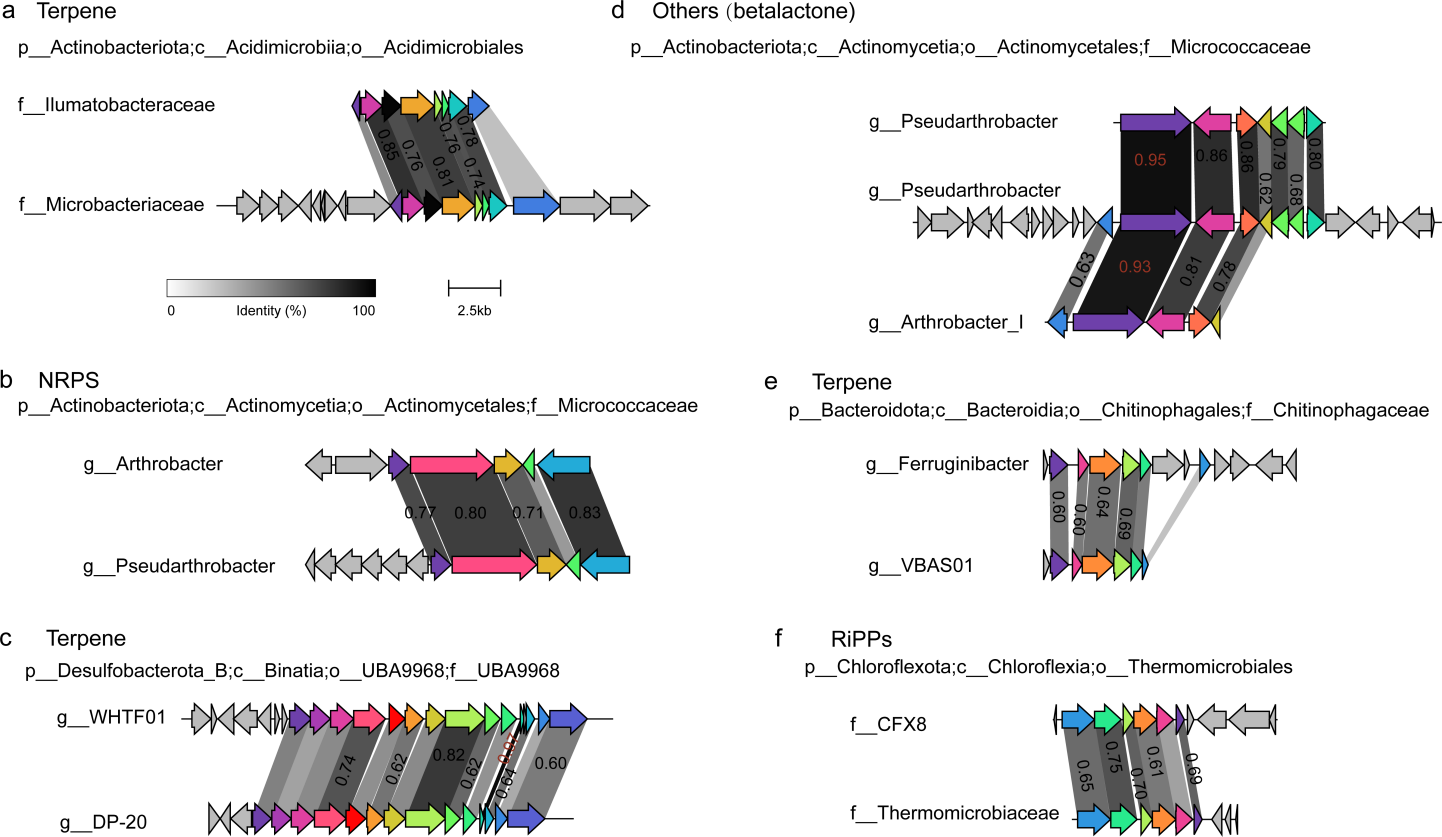
The GCFs shared by different bacterial genera or families. The BGCs were clustered into each GCF by BIGSCAPE with a default threshold of 0.3. The grayscale links between the genes of pairwise BGCs represent sequence identity. A link was shown if the sequence identity was >30%, and the number was shown if the sequence identity was >60%. The BGCs were annotated by antiSMASH and visualized by Clinker (33).

However, we also found that six GCFs were shared by different bacterial genera or even families (Fig. 3). These GCFs contain three terpenes, one NRPS, one RiPP and one betalactone, in which most of the pairwise BGCs have at least one biosynthetic gene with >80% sequence identity. For example, in the terpene GCF shared by two different families, i.e., *Ilumatobacteraceae* and *Microbacteriaceae*, four biosynthetic genes of the pairwise BGCs have >70% sequence identity and two biosynthetic genes have >80% sequence identity (Fig. 3a). The genera *Pseudarthrobacter* and *Arthrobacter* shared one GCF, in which one of their biosynthetic genes had >90% sequence identity (Fig. 3d). This observation implies a history of horizontal transfer of BGCs between different soil bacterial groups, which is essential for bacteria to rapidly enhance their competitive advantage and environmental fitness (4, 32).

Further genome mining revealed 25 bacterial genomes as BGC-rich species (each with >10 BGCs) within the phyla Actinobacteriota, Acidobacteriota, Myxococcota, and γ-Proteobacteria (Fig. 2a). More than half (13 genomes) of these BGC-rich species were annotated as *Actinomycetia* under phylum Actinobacteriota, including the well-known genera *Streptomyces*, *Mycobacterium,* and *Nocardia* (4, 34). Although these species have been exploited to produce a wide range of medicinal compounds, such as the antibiotics streptomycin and chloramphenicol and the anticancer drug doxorubicin (34), our analysis confirmed that nearly 70% of BGCs in Actinobacteriota had no representatives in the MiBIG database (Fig. 2c). This suggests that there is still much more room for the discovery of new natural products from this biosynthetically versatile phylum, as recently exemplified by the discovery of novel T2PKS (30). In addition, we also recovered some bacterial genera that were recently noticed for their enormous biosynthetic potential, such as the uncultivated genera UBA5704 (26, 35) in phylum Acidobacteriota. The BGCs encoded in UBA5704 genomes did not form any GCFs with BGCs in the MiBIG (Data set S5), indicating the massive potential of this uncultivated taxon for the discovery of new natural products. Notably, most of the BGC-rich genomes (21 genomes) failed to be classified at the species level, including a previously unexplored BGC-rich bacterial lineage (24 BGCs) belonging to an uncharacterized candidate family under order *Mycobacteriales* (Data set S3). Altogether, the results provide the first access to previously uncharted taxonomy information of BGCs in agricultural soils of China.

### Biotic and abiotic factors jointly shape the biogeography of biosynthetic potentials

**Fig 4:**
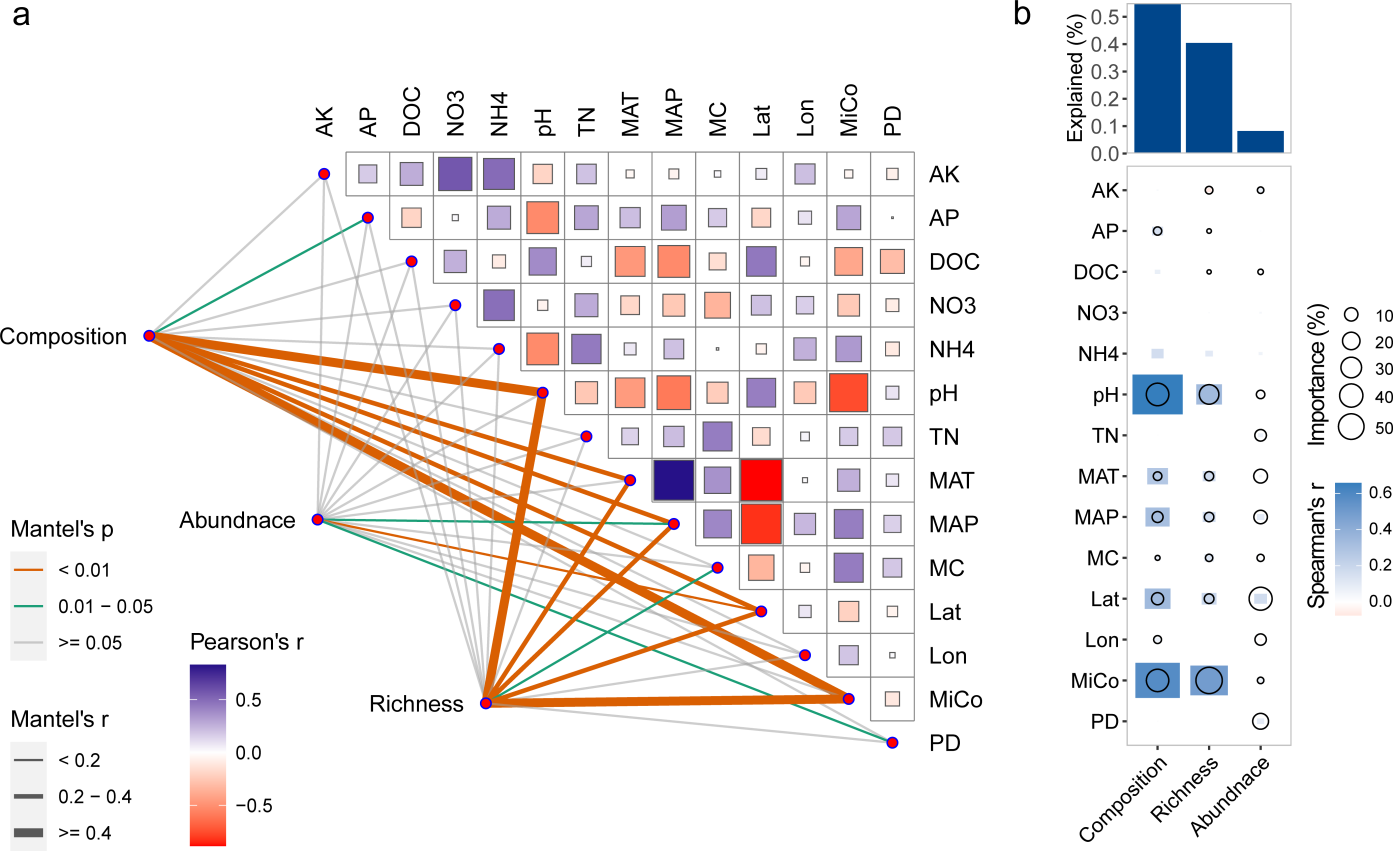
The biotic and abiotic drivers of BGC profiles in agricultural soil microbiomes. (**a**) Mantel test analysis showing the correlation of biotic and abiotic variables with BGC composition, abundance, and richness. (**b**) Contributions of variables to dissimilarities of BGC composition, abundance, and richness based on correlation and best multiple regression model. Circle size represents the variable importance (proportion of explained variability calculated by multiple regression modeling and variance decomposition analysis). The heatmap colors represent Spearman correlations between differences in variables and BGC composition. MC, moisture content; MiCo, microbial composition; PD, microbial phylogenetic diversity.

The BGC profiles have shown clear distinction among agricultural soils (Fig. 1b; Fig. S2). However, little is known about what abiotic (or environmental) and biotic factors contribute to the divergence of BGC profiles. To address this important question, a series of statistical analyses were performed. Mantel test analysis revealed that microbial composition, soil pH, mean annual precipitation (MAP), mean annual temperature (MAT), and latitude significantly contributed to the variance of BGC composition (*P* < 0.05, Fig. 4a). BGC richness was greatly driven by microbial composition and several environmental variables, such as pH, MAT, MAP, latitude, and soil moisture content (Fig. 4a). Moreover, we found that BGC abundance was rarely influenced by the examined 14 variables, while some BGC Classes (e.g., PKSI, Hybrids and Terpene) were significantly influenced by some variables, such as soil pH or MAP (Fig. S7). Furthermore, multiple regression modeling revealed that the biotic and abiotic factors jointly explained 55.0%, 40.4%, and 8.2% of the variations of BGC composition, richness, and abundance, respectively (Fig. 4b).

**Fig 5:**
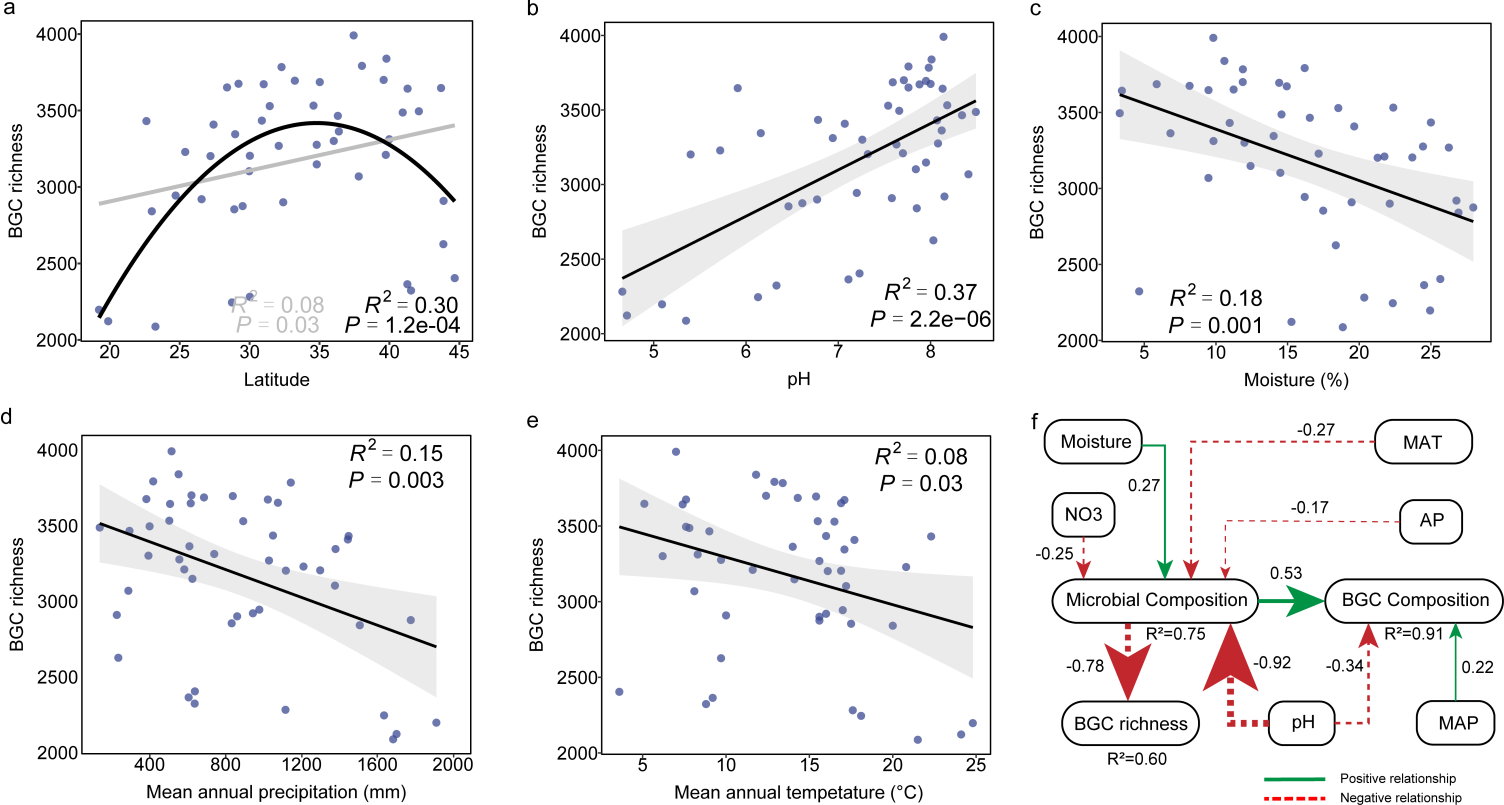
The environmental drivers of BGC richness in agricultural soil microbiomes. (**a**) Latitudinal distribution of BGC richness. (**b-e**) The Spearman correlation of BGC richness with soil pH, soil moisture, mean annual precipitation, and mean annual temperature, respectively. (**f**) structural equation modeling (SEM) of BGC richness and composition. Numbers near the pathway arrow indicate the standard path coefficients (β). The arrow width is proportional to the strength of the relationship. R^2^ represents the proportion of variance explained for every dependent variable. The goodness of fit was acceptable: Model χ2 = 11.5, df = 11, GFI = 0.986, CFI = 0.998; root mean square error of approximation (RMSEA) = 0.03.

We further pinpointed the specific relationships between the main environmental variables and BGC richness. The results showed that BGC richness significantly increased toward latitudes, peaked at mid-latitudes, and then showed a declining trend with further elevated latitudes (R^2^ = 0.30, *P* = 1.2e-04) (Fig. 5a). The same nonmonotonic pattern was also observed for functional gene diversity in global topsoil microbiomes (36). Moreover, of the eight edaphic and two climatic variables examined, BGC richness significantly increased from acidic to alkaline soils, with the highest fit coefficient with soil pH (R^2^ = 0.37, *P* = 2.2e-06) (Fig. 5b). A similar result was also identified by an amplicon-based survey of bacterial secondary metabolism in soils of the United States (17), indicating universal consistency in the positive relationship between BGC richness and soil pH across different continental regions.

In contrast to soil pH, soil moisture (R^2^ = 0.18, *P* = 0.001, Fig. 5c), MAP (R^2^ = 0.15, *P* = 0.003, Fig. 5d) and MAT (R^2^ = 0.08, *P* = 0.03, Fig. 5e) were negatively correlated with BGC richness. Previous reports indicated that low-moisture soil environments may lead to increased pressures on nutrient acquisition and/or other means of competition, leading to an increase in BGC richness (14, 17). In this study, microbial co-occurrence networks showed that the proportion of negative edges (indicating microbial competition (37–39) increased from 2.4% in high-moisture soils (20.4% to 28.0%) to 4.5% in low-moisture soils (3.3% to 12.0%) (Table S3), revealing an increased intensity of competition with reduced soil moisture. Therefore, we postulated that microbes with diversified BGCs were enriched in low-moisture soils, as they could mediate a high intensity of antagonistic or competitive biological interactions to outcompete others. Consistent with the above results, structural equation modeling also revealed the strong direct effect of soil pH (standardized path coefficient, *β* = −0.92, *P* < 0.001) and moisture (*β* = 0.27, *P* < 0.01) on the microbial community structure, which in turn significantly affected the richness of soil BGCs (*β* = −0.78, *P* < 0.001) (Fig. 5f).

### BGC-inferred biotic interactions are correlated with soil microbiota assembly

Environmental characteristics of soils can shape specific ecological niches to determine what species of microbes (the potential hosts of BGCs) can survive there. This largely determines the BGC profile differences, as we revealed in the last section. However, the mechanism by which microbes in the same ecological niche are organized into microbial communities remains unclear. In this context, secondary metabolites produced by BGC-carrying microbes may mediate microbiota-specific biotic interaction networks to structure the environmental microbiota, considering that BGC-inferred biotic interactions (e.g., competition, predation, antagonism, and mutualism) within microbes have been widely recognized (40–42). Given the above knowledge, we hypothesized that BGC-rich taxa could play key roles in structuring microbial communities, and that the BGC profiles of soil microbiomes would mediate community assembly.

**Fig 6:**
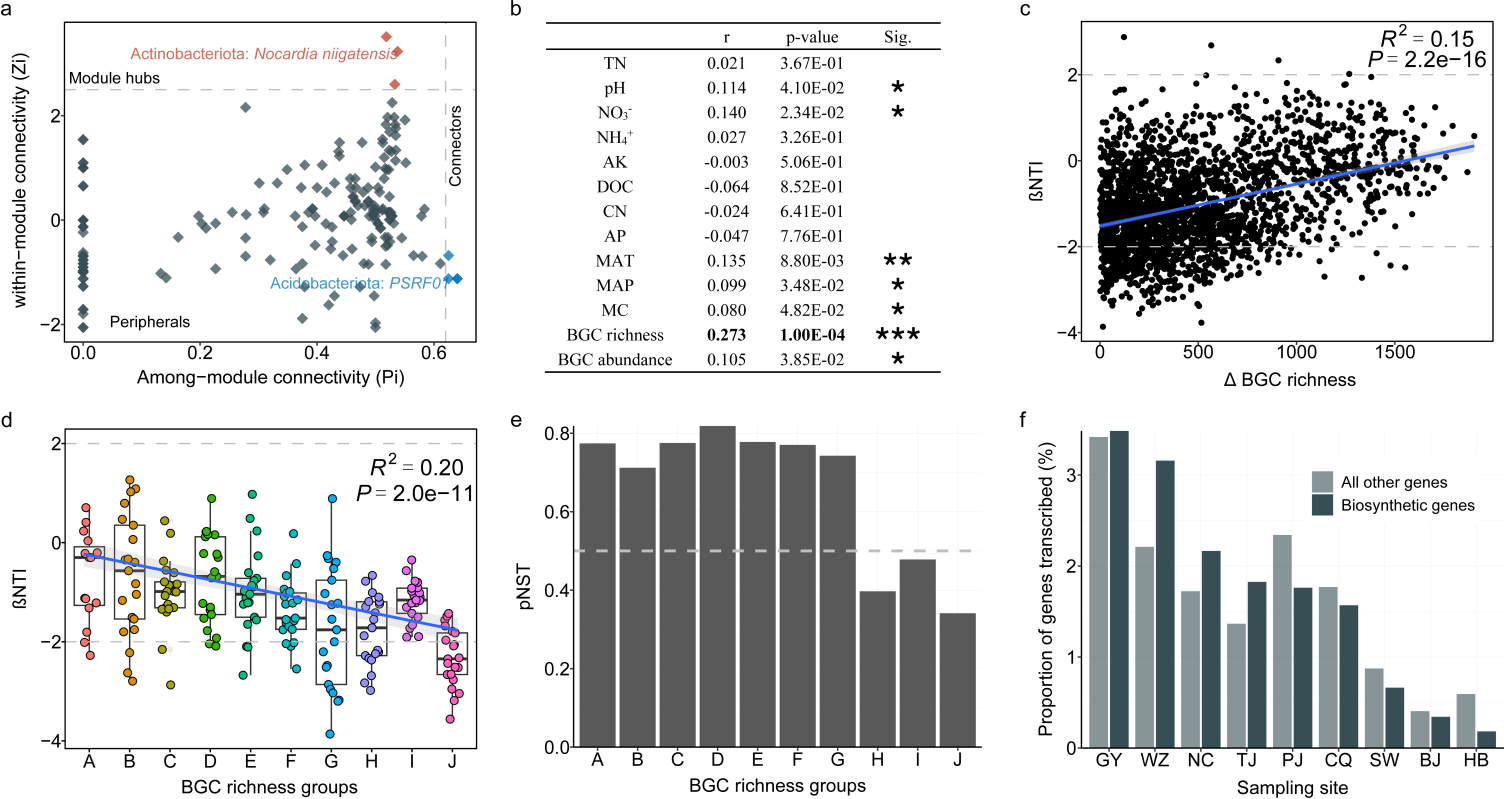
The influence of BGC richness on microbial communities. (**a**) The keystone taxa identified in the co-occurrence network. Each node represents an MAG. The node is defined as module hub if its within-module connectivity (Zi) ≥ 2.5 and among-module connectivity (Pi) < 0.62, as connector if its Pi ≥ 0.62 and Zi < 2.5, as network hub if its Zi ≥ 2.5 and Pi ≥ 0.62, and all other nodes are identified as peripherals. (**b**) Mantel tests of abiotic and biotic factors against the phylogenetic turnover (β-nearest taxon index) of microbial communities. (**c**) The relationship between the β-nearest taxon index (βNTI) and differences (Δ) in BGC richness. (**d**) Patterns of βNTI across different groups of BGC richness. The samples were artificially separated into 10 groups (**a-j**) with an ascending BGC richness. Each group contains seven samples, but one sample with abnormally low BGC richness was excluded from the A group. The BGC richness increased from 2,087 in the A group to 3,991 in the J group. The relationship was estimated by linear least-squares regression analysis. (**e**) The normalized stochasticity ratio (NST) based on the phylogenetic beta diversity index across the different categories of BGC richness, and the group separation is identical to Fig. 6d. The assembly is dominated by the stochastic process if pNST >50%. It is dominated by the deterministic process if pNST <50%. (**f**) The proportion of biosynthetic genes transcribed in different samples.

To test this hypothesis, we constructed a microbial co-occurrence network of the recovered 449 bacterial genomes. Topological analysis revealed two BGC-rich species, i.e., *Nocardia niigatensis* from Actinobacteriota and *PSRF01* from Acidobacteriota, as the module hub and connector, respectively (Fig. 6a), indicating their essential role as keystone species in microbial communities. The MAG of *N. niigatensis* and the MAG of *PSRF01* contains 48 and 21 BGCs, respectively, which could potentially synthesize a wide range of secondary metabolites. These uncharacterized compounds may play important functions in mediating microbial interactions, conferring their producers as the keystone species in microbial communities. However, only two BGCs (one RiPP from *PSRF01* and one NRPS from *N. niigatensis*) were mapped with mRNA reads at the time point of sampling. Therefore, future controlled time-series experiments coupled with metatranscriptomics and metabonomics should be conducted to identify the active ecological functions of secondary metabolites encoded by these BGCs.

In addition to the key roles of BGC-rich taxa in the microbial community, we also observed the significant association of biotic-inferred interactions with microbial community assembly. Mantel test results showed that BGC richness was the best predictor (r = 0.27, *P* = 1.0e-04) of microbial phylogenetic turnover (quantified by β-nearest taxon index, βNTI) when compared with the other 12 measured biotic and abiotic variables (r = 0.08 to 0.14, Fig. 6b). The βNTI values were positively correlated with differences in BGC richness between samples (R^2^ = 0.15, *P* = 2.2e-16, Fig. 6c), indicating the strong influence of BGC richness on soil microbiota assembly. Furthermore, with an increasing level of BGC richness (from 2,087 to 3,991), the relative importance of deterministic assembly greatly strengthened (from 13.3% to 66.7%), whereas that of stochastic processes largely weakened (Fig. 6d). This result generally agreed with the normalized stochasticity ratio (NST) estimation, in which the relative importance of stochastic processes decreased in groups with high BGC richness (Fig. 6e). To check whether the BGCs are actively expressed in the *in situ* soil environment, we investigated the transcripts of BGCs in nine representative soil samples. On average, 1.7% of the biosynthetic genes were transcribed, which was slightly higher than the transcribed proportion of all other microbial genes (1.6%) (Fig. 6f), suggesting the active expressions of BGCs in the environment. Collectively, these results are consistent with our hypothesis that BGC-inferred biotic interactions play dominant roles in structuring soil microbiota.

## DISCUSSION

Understanding how BGCs are distributed in environmental microbiomes and what factors drive their biogeographic distribution is of general interest from two-tier perspectives, i.e., bioactive compound discovery and microbial ecology. By conducting metagenomic analyses of soil microbiomes on a continental scale, this study revealed the enormous diversity and high novelty of bacterial BGCs that support the great potential for the discovery of previously undescribed chemistry in Chinese agricultural soils, as recently implicated in grassland soils of the United States (9) and Antarctic soil (11). The most abundant novel BGCs in this study corresponded to the predicted NRPS and PKS, which are usually responsible for producing diverse bioactive natural products, including antibiotics and anticancer agents (43). Metagenomic mining of BGCs, coupled with functional validation based on heterogeneous expression or other advanced functional genomics technologies, will make agricultural soils a promising field for metagenomics-guided discovery of new bioactive compounds from numerous uncultivated microorganisms.

Moreover, we found that the geographic distribution of soil bacterial BGCs was strongly associated with soil pH, moisture and MAT. Further SEM analysis revealed these key environmental factors directly affected the microbial community structure, which in turn significantly affected the richness of soil BGCs. For example, soil pH exhibited a significant and positive correlation (R^2^ = 0.13, *P* = 0.006) with *Actinomycetia* abundance (Fig. S8). Considering the fact that more than half of the BGC-rich clades identified in this study (Fig. 2a) were predicted to be hosted by *Actinomycetia*, which are well-known as extremely versatile producers of bioactive natural products (4), the pH-induced decrease in soil *Actinomycetia* abundance in acidic soil may prominently contribute to the reduction of BGC richness. Furthermore, this novel view of the continental-scale patterns of BGC distributions indicates that soil acidification and global climate change (e.g., shifts in precipitation and temperature) may substantially affect the biosynthetic potential of the soil microbiome. Specifically, the soil acidification resulting from acid rain and ammonium-based fertilization may substantially decrease BGC richness by inhibiting the proliferation of BGC-rich bacteria (e.g., *Actinomycetia*), which can, in turn, cause negative impacts on the soil ecological balance and discovery of novel medicines.

Understanding the mechanisms controlling community diversity and assemblage is a central issue in microbial ecology (44). Many studies have explicitly deciphered the impacts of abiotic factors (e.g., spatial scale, pH, MAT, and MAP) on the assembly processes of microbial communities (45–47). However, we revealed that compared with external abiotic drivers, BGC richness was the best predictor of microbial phylogenetic turnover, indicating BGC-inferred biotic interactions are a dominant deterministic factor and internal driver in shaping microbial communities. Because a microbial community with higher BGC richness is expected to produce more diversified secondary metabolites to strongly mediate community-wide biotic interactions (i.e., competition, antagonism, and mutualism), the phylogenetic turnover between bacterial communities with high BGC richness is, therefore, more deterministically driven. In contrast, biotic interactions are likely to be very weak in a microbial community with low BGC richness, allowing for an elevated stochastic influx of new species (e.g., immigration) into the community (45). Moreover, although the essential roles of secondary metabolites in microbial ecology have been widely acknowledged over the last few decades (40, 41, 48), this study further identified the keystone role of BGC-rich taxa in microbial communities and uncovered and quantified the strong association of BGC profiles with microbial phylogenetic turnover. Importantly, considering the critical roles of microorganisms in soil ecosystems (49), revealing the intrinsic mechanisms and ecological rules guiding assembly processes and species coexistence could greatly benefit the management of microbial communities to enhance agricultural production in response to environmental changes (50). Therefore, additional efforts should be put to further validate the ecological roles of BGCs and BGC-rich species in microbial communities.

Until now, the specific functions of the enormous secondary metabolites encoded by microbial BGCs in nature have rarely been characterized and largely underexploited, so there is still an enthusiastic need for tremendous efforts to recover the specific roles of diverse secondary metabolisms in the environment, which is critical to enhancing our understanding of the ecological roles and biotechnological importance of bacterial secondary metabolites.

## MATERIALS AND METHODS

### Soil sampling, biogeochemical analysis, and data collection

Soil samples were collected from agricultural land across 31 provincial-level administrative regions of mainland China from April 13th–25th, 2021, covering all climate zones, including temperate, subtropical, tropical, and highland climate zones. Fifty sampling sites were chosen with the consideration of geographical representation and vegetation types (e.g., maize, rice, wheat, and oilseed rape). For each site, five soil cores obtained at a depth of 0–15 cm were combined. Additionally, we collected samples at depths of 15–30 cm and 30–45 cm from ten of the sampling sites.

Standard test methods were employed to measure soil pH, moisture content (MC), dissolved organic carbon (DOC), total nitrogen (TN), nitrate nitrogen (NO_3_^-^), ammonium-nitrogen (NH_4_^+^), available phosphorus (AP), and available potassium (AK), as previously described (46, 51). We obtained climate data, including mean annual temperature and mean annual precipitation, from the WorldClim database (www.worldclim.org).

### DNA extraction, metagenomic sequencing, and 16S rRNA gene amplicon sequencing

For each soil sample, DNA was extracted using the FastDNA Spin Kit for Soil (MP Biomedicals, USA). The metagenomic libraries were then prepared and sequenced on the Illumina NovaSeq 6,000 platform using a paired-end 150 bp strategy at the Novogene Corporation (Beijing, China). In total, the metagenomic sequencing produced 1.33 Tb nucleic acid bases across all 70 samples, corresponding to 8.84 × 10^9^ reads with an average read count of 1.26 × 10^8^ reads per sample. The metagenomic data covered 24.5%-53.6% of the soil microbial diversity (Fig. S1). One metagenome was excluded from the correlation analysis due to its abnormal coverage (84.8%). The V4-V5 hypervariable regions of prokaryotic 16S rRNA genes were amplified using the forward primer 515F and reverse primer 926R (primer sequence), and the amplicons were then sequenced on the Illumina NovaSeq 6,000 platform (PE250) at the Magigene Biotechnology Corporation (Guangzhou, China). The amplicon sequencing data were then processed as described in our previous study (52).

### Metagenomic assembly and binning

For each metagenomic dataset, raw sequencing reads were processed with Fastp (v0.23.1) (53) to remove Illumina adaptors, low-quality reads, and duplicate reads. Deduplication could substantially improve the assembly and binning results of soil metagenomes, as evaluated by our previous study (54). Clean reads of each sample were then individually *de novo* assembled using MetaSPAdes (v3.15.4) (55).

The coverage profiles of each assembly were generated by mapping clean reads from every sample using BWA (56). The scaffolds of each assembly were subsequently clustered into genome bins informed by the coverage profiles using three different binning software programs (i.e., MetaBAT2 (57), MaxBin2 (58), and CONCOCT (59) in the MetaWRAP pipeline (v1.3.0) (60) with parameter -l 2,000. High-quality draft genomes of each sample were then extracted from the above-generated bins using the bin refinement module in MetaWRAP. In another strategy, the coverage profile of each assembly was also generated by only mapping clean reads from its sample and was used to inform binning as described above. The bins with an overall quality of >50% (completeness – 5 × contamination) were considered eligible ones. Finally, 701 and 512 bins were obtained using cross-sample and individual-sample binning strategies, respectively (Fig. S1).

Although the binning yields of cross-sample binning were significantly higher than those of individual-sample binning, each strategy could recover some unique bins. To obtain representative genomes from the soil metagenomes as much as possible, the bins from both strategies were combined and dereplicated using dRep (v3.0.0) (61) with a 95% ANI threshold, finally resulting in 510 species-level representative metagenome-assembled genomes (MAGs). MAGs were taxonomically assigned using the classify_wf module of gtdbtk (v2.1.1) (62) and were classified into 449 bacterial MAGs and 61 archaeal MAGs. Phylogenetic analysis of MAGs was conducted with the gtdbtk infer module based on a set of 120 bacterial or 53 archaeal-specific marker genes from GTDB (62), and the phylogenetic trees were visualized in iTOL (63). The abundance of MAGs in each sample was quantified using CoverM (v0.6.1) (https://github.com/wwood/CoverM). The co-occurrence network of soil bacterial genomes and global network properties were calculated using the Molecular Ecological Network Analysis Pipeline (64).

### Biosynthetic gene cluster (BGC) annotation and analysis

Contigs longer than 2 kb were analyzed using antiSMASH (v6.1.1) (25) with default parameters to identify BGCs. The BGCs were further clustered into gene cluster families (GCFs) based on the pairwise BGC distances (0.3), which were calculated as the weighted combination of the Jaccard Index, adjacency index, and domain sequence similarity using biosynthesis-related gene similarity clustering and prospecting engine (BiG-SCAPE) software with the mode auto (65). The longest BGC within each GCF was chosen as the representative. To estimate BGC novelty, BiG-SLiCE (66) was used to calculate the distances between the BGCs of this study and the BGCs of the computationally predicted BiG-FAM (28) and experimentally validated MIBiG (29) database, which had been computed using t = 900 as a threshold. The resulting distance indicates the degree to which a given BGC differs from previously computed GCFs, with a higher distance indicating higher novelty.

### BGC abundance and diversity calculation

To calculate the relative abundance of BGCs, the contigs carrying the representative BGCs were merged to build an index file, and the clean reads from each sample were mapped to the index file using Bowtie2 (v2.3.4.1) (67). After obtaining the sorted BAM file of each sample, the bedcov function in SAMtools (v1.15.1) (68) was used to extract mapping information of BGCs informed by the bed file that had the location information of BGCs on the contigs. The relative abundance of each BGC was calculated as the amount of reads mapping to the BGC normalized by BGC length and the size of the metagenome, as shown in the following equation:


$$\text{ Abundance (coverage, }\times /Gb)=\frac{N_{\text{mapped reads }}\times L_{\text{reads }}/L_{BGC}}{S}$$

where *N*_mapped reads_ is the number of reads mapped to one specific biosynthetic reference gene; *L*_reads_ is the sequence read length; *L*_BGC_ is the nucleotide length of the corresponding BGC; and *S* is the size of the metagenomic data (Gb). The BGC richness of each sample was estimated using the estimateR function of the vegan package in R (69).

### Metatranscriptomic analysis

To check whether the BGCs are actively expressed in the *in situ* soil environment, we selected 9 out of the 70 samples for metatranscriptomics analysis with the consideration of the geographical representation and vegetation types (i.e., maize, rice, wheat, oilseed rape, and others). Total RNA was extracted from each of the nine soil samples using the RNA PowerSoil Total RNA Isolation Kit (MoBio, USA). After the removal of ribosomal RNA for microbial RNA, Illumina’s TruSeq Stranded mRNA LT Sample Prep Kit (Illumina, USA) was used for reverse transcription as well as macrotranscriptome birdshot sequencing library construction. Each library was sequenced by the Illumina NovaSeq platform (Illumina, USA) with the PE150 strategy at Personal Biotechnology (Shanghai, China). Transcripts were quality-controlled using trim_galore (v0.6.7) (Babraham Bioinformatics - Trim Galore!). On average, metatranscriptomic sequencing produced 7.4 × 10^7^ clean reads per sample. The RNA reads were then mapped to target genes using hisat2 (v2.2.1) (70). The generated SAM files were transformed into BAM files and then sorted using SAMtools (v1.15.1) (68). The reads mapped to each gene were counted using HTSeq-count (v2.0.2) (71). Transcripts that have at least five counts were reported and included in downstream analyses in order to exclude low levels of read mapping, as in the previous study (13).

### Microbial community assembly mechanism analysis

The assembly process of microbial communities was identified by using the bNTIn.p module in iCAMP (72) with a parallel computing mode. A beta nearest taxon index (βNTI) value of less than −2 indicates significantly less phylogenetic turnover than expected (i.e., homogeneous selection), while a βNTI value of more than two indicates significantly more phylogenetic turnover than expected (i.e., variable selection). A |βNTI| < 2 indicates the dominance of stochastic processes. Furthermore, the major biotic and abiotic factors that influenced the assembly processes of soil microbial communities were investigated. Variation in community assembly processes along the gradients of the major factors was assessed using the Mantel test that correlated the βNTI values with the Euclidean distance matrices of each factor. Furthermore, the normalized stochasticity ratio (NST) was used to quantify the relative importance of deterministic and stochastic processes in the microbial community assembly (73). The NST index based on the phylogenetic beta diversity index (pNST) was calculated using the null model algorithm PF (fixed data richness and proportional taxa occurrence frequency) as described in a previous study (74). The NST index of 50% was adopted as the boundary point between more deterministic (<50%) and more stochastic (>50%) assembly of microbial communities. In addition, microbial co-occurrence network analysis was performed to predict the intensity and role of biotic interactions (e.g., competition) in the community assembly using the ‘Co-occurrence_network.R’ script of MbioAssy1.0 (39, 75).

### Statistical analysis

To visualize the variation in BGC composition across samples, the non-metric multidimensional scaling (NMDS) analysis was conducted on the BGC Bray‒Curtis dissimilarity matrix using the metaMDS function of the vegan package (69) in R. The significance test of pairwise comparison of each vegetation type was conducted using pairwiseAdonis in R. OTU richness was estimated using the estimateR function of the vegan package in R, and phylogenetic diversity was calculated using the pd function in the picante R package (76).

Structural equation modeling (SEM) was used to explore the direct and indirect relationships among environmental variables, microbial communities, and BGC compositions. The microbial community composition was represented by PC1 of the principal coordinate analysis based on the Bray–Curtis distance. Initially, we constructed a hypothesized model that included all reasonable pathways. Then, we sequentially pruned the nonsignificant pathways or added new pathways based on residual correlations until the model showed sufficient fitting with *P* values of the χ^2^ test larger than 0.05 (i.e., the predicted model and observed data were not significantly different), and the root mean square error of approximation (RMSE) was less than 0.08. The SEM-related analysis was performed using the lavaan R package (77).

## Data Availability

The raw sequencing data, including 16S rRNA gene amplicon sequencing, metagenomic sequencing data, and metatranscriptomic sequencing data, are accessible in the China National GeneBank DataBase under the accession no. CNP0004176. The R codes for the statistical analyses are freely available at INFINITY1993/BGCs-in-nationwide-agricultual-soils (github.com), and the R and python codes used for microbial community assembly mechanism and co-occurrence network analyses are freely available at emblab-westlake/MbioAssy (github.com).

## SUPPLEMENTAL MATERIAL

Supplemental Material msystems.01263-23-s0001.pdf Supplemental figures and tables.

Supplemental Data Set msystems.01263-23-s0002.xlsx Data Set S1

ASM does not own the copyrights to Supplemental Material that may be linked to, or accessed through, an article. The authors have granted ASM a non-exclusive, world-wide license to publish the Supplemental Material files. Please contact the corresponding author directly for reuse.
