# Supplementary material for: Diversity and distribution of biosynthetic gene clusters in agricultural soil microbiomes
Source: mSystems. 2024 Mar 12;9(4):e01263-23. doi: 10.1128/msystems.01263-23 (PMC11019929; doi:10.1128/msystems.01263-23)
Supplement: Supplemental Material — Supplemental figures and tables. [file msystems.01263-23-s0001.pdf]

SUPPLEMENTARY MATERIAL for

Diversity and Distribution of Biosynthetic Gene Clusters in Agricultural Soil Microbiomes

1. Supplementary figures

Fig. S1. The workflow of bioinformatic analysis of China agricultural soil metagenomes.

Fig. S2. The BGC composition (a) and richness (b) in different sampling sites.

Fig. S3. The CLFs of 17 type II PKS (T2PKS) recovered from the agricultural soils.

Fig. S4. The 61 species-level representatives of archaeal MAGs recovered from the agricultural soil metagenomes.

Fig. S5. The detection frequency of the 449 bacterial MAGs across the 70 soil samples.

Fig. S6. Percentage of BGC classes within different taxonomic phylum groups.

Fig. S7. Mantel test analysis showing the correlation of biotic and abiotic variables with BGC composition, abundance and richness.

Fig. S8. The correlation between the abundance of *Actinomycetia* and soil pH.

2. Supplementary tables

Table S1. The PERMANOVA test of pairwise vegetation types.

Table S2. The significance test of partial RDA-based variance partition analysis.

Table S3. The network topology of samples grouped by soil moisture or BGC richness.

1. Supplementary figures

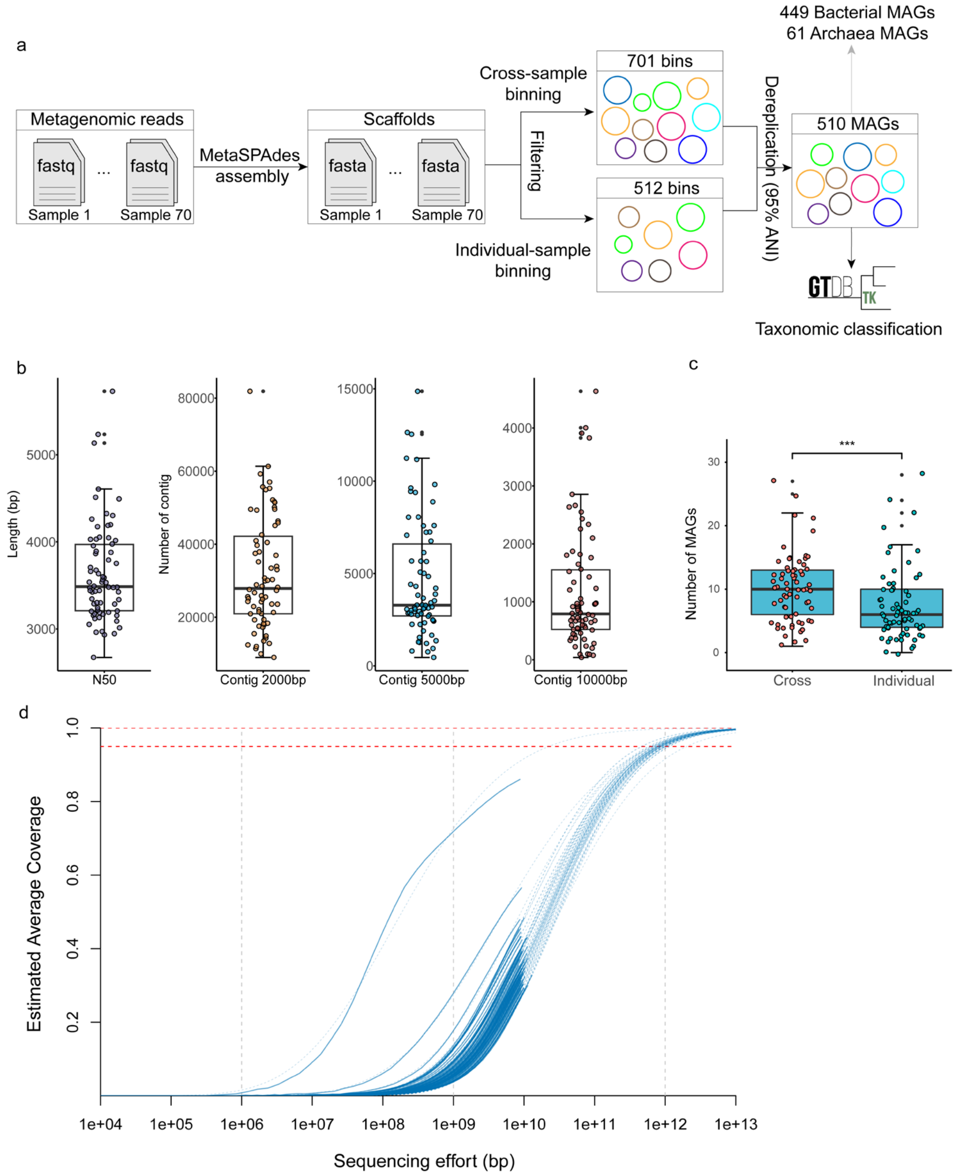

Fig. S1. The workflow of bioinformatic analysis of China agricultural soil metagenomes. **a.** The metagenomic assembly and binning strategies used in this study. **b.** A summary of the 70 metagenomic assemblies, e.g., the length of N50, the number of contig > 2000bp in size and so on. **c.** Two binning were adopted, i.e., cross-sample and individual sample binning, in this study. Finally, 701 and 512 bins were obtained by cross-sample and individual-sample binning strategies, respectively. Although the binning yields of cross-sample binning were significantly higher than that of individual-sample binning, each strategy could recover some unique bins. To obtain representative genomes from the soil metagenomes as much as possible, the bins from both strategies were combined and dereplicated with a 95% ANI threshold, generating 510 species-level representative metagenome-assembled genomes (MAGs). **d**. Nonpareil estimates of sequence coverage for the 70 metagenomes studied.

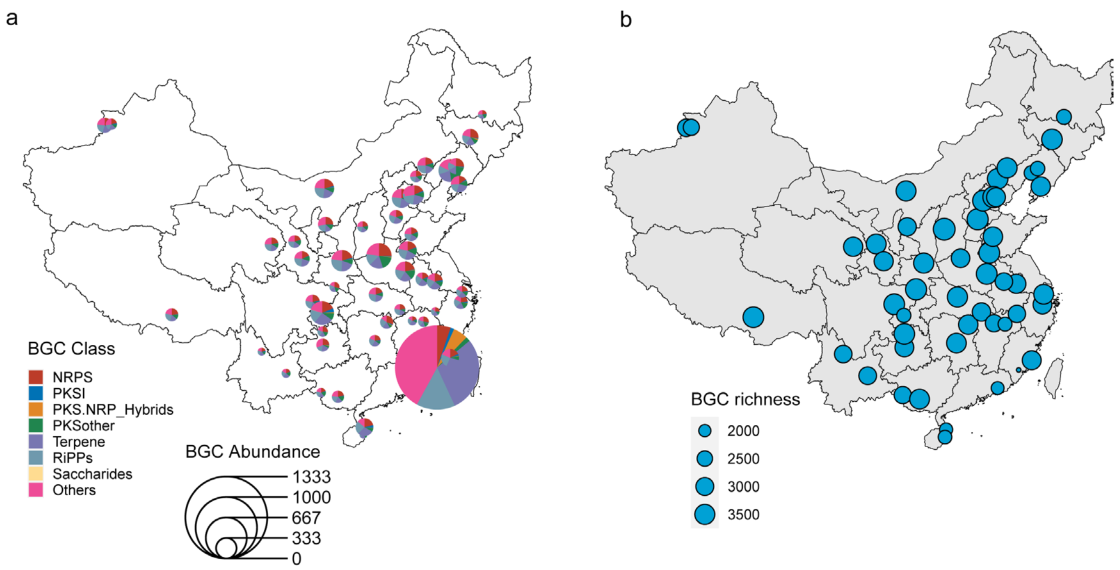

Fig. S2. The BGC composition (a) and richness (b) in different sampling sites.

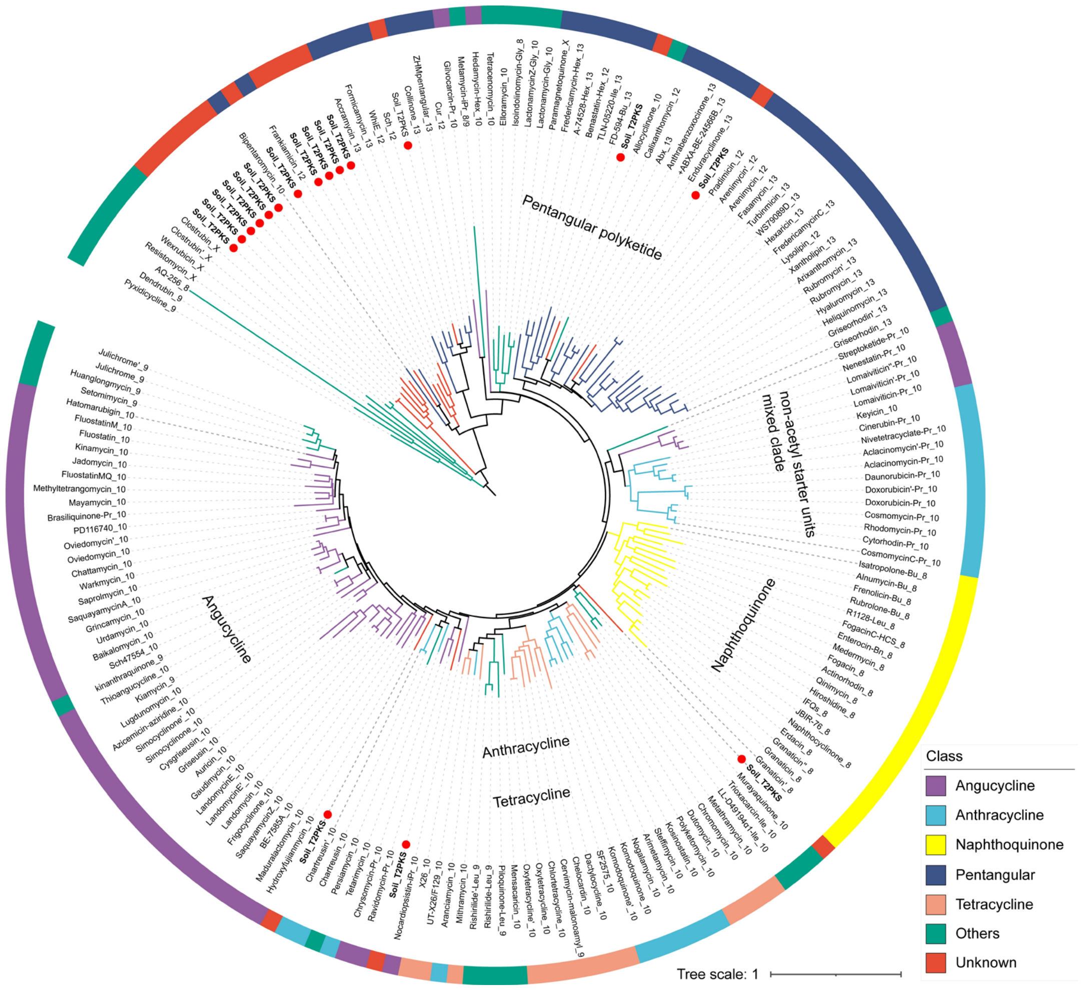

Fig. S3. The CLFs of 17 type II PKS (T2PKS) recovered from the agricultural soils. These CLFs all have a 0.37-0.79 similarity (<0.88) with the CLFs of known T2PKS curated by a recent study [1], in which they found that CLF as a marker can best represent the chemical product differences, and a threshold of CLF identity of 0.88 worked well to evaluate whether the products of T2PKS are identical to known ones or not.

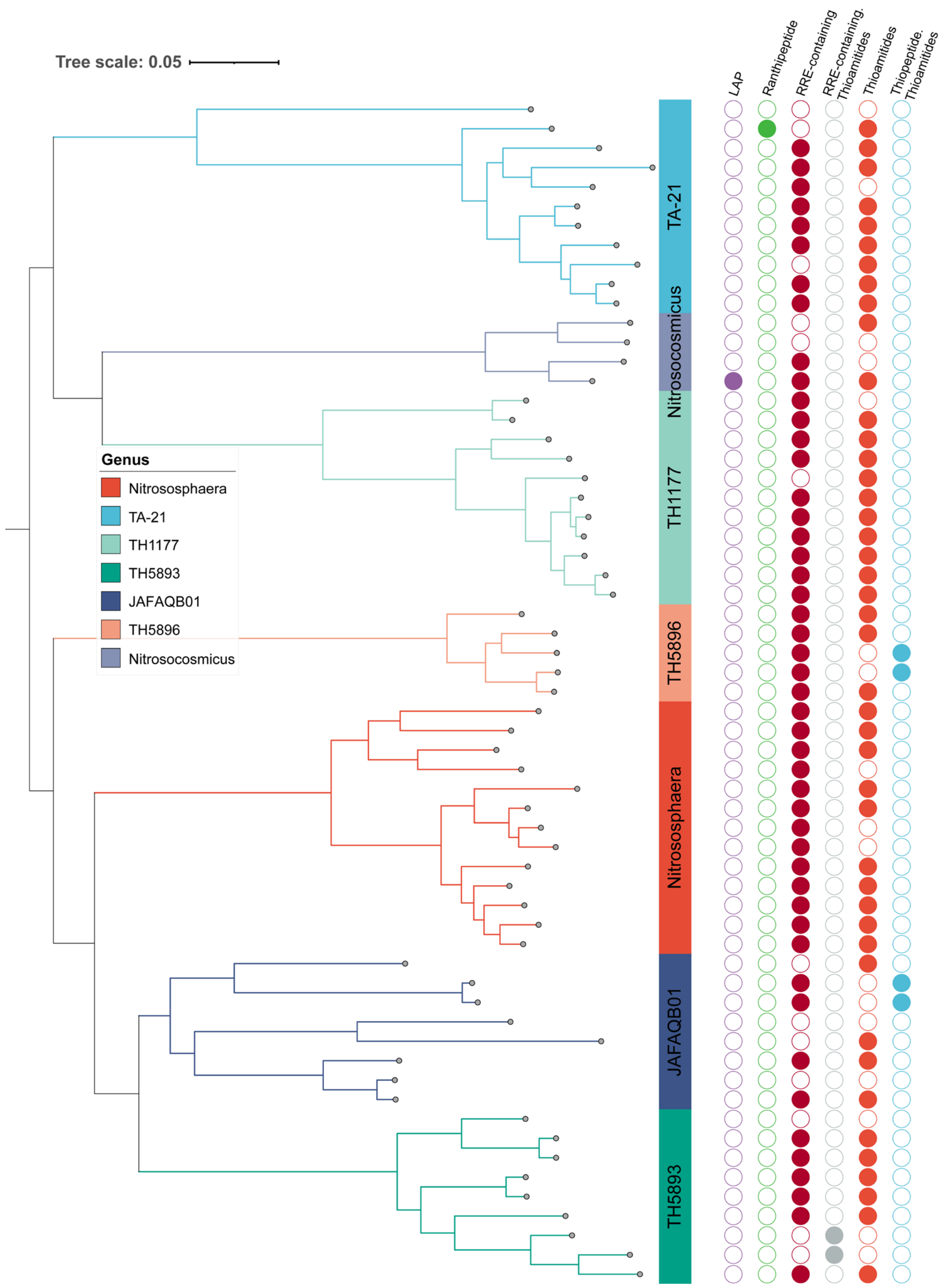

Fig. S4. The 61 species-level representatives of archaeal MAGs recovered from the agricultural soil metagenomes. The phylogenetic tree was constructed based on 53 archaeal specific marker genes using GTDBTk and visualized in iTOL. The filled circles indicate the presence of BGCs in the archaeal genomes. The archaeal MAGs are all from the family *Nitrososphaeraceae*, and they carry up to three BGCs belonging to the RiPPs or Others classes.

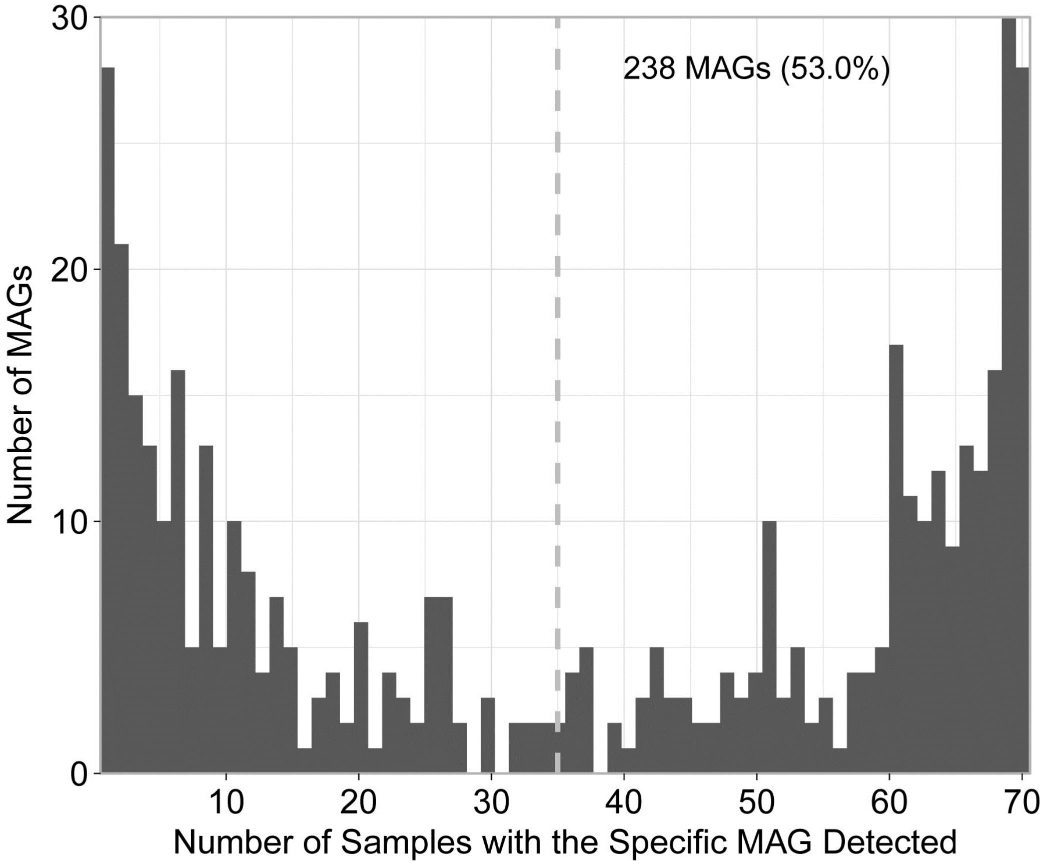

Fig. S5. The detection frequency of the 449 bacterial MAGs across the 70 soil samples.

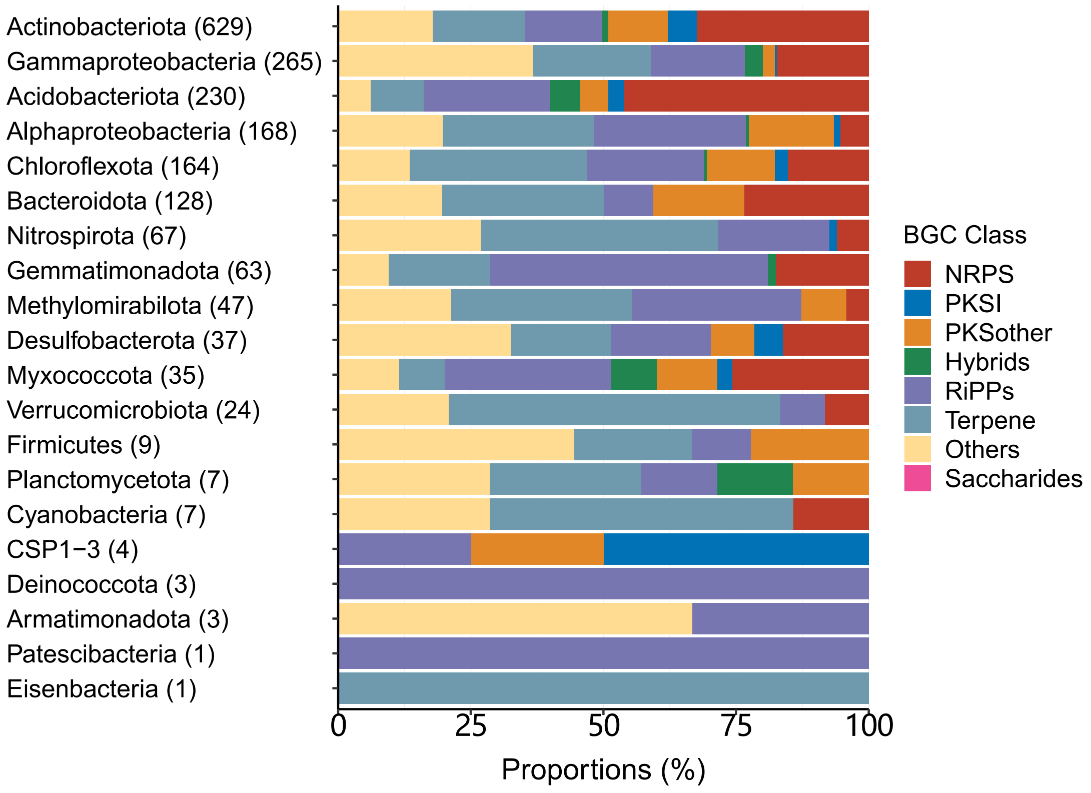

Fig. S6. Percentage of BGC classes within different taxonomic phylum groups. Total number of BGCs per group is indicated in parentheses.

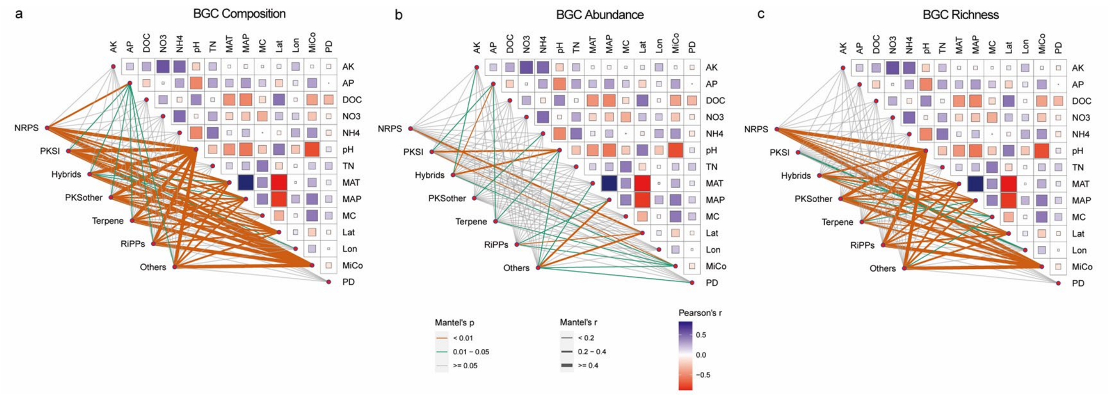

Fig. S7. Mantel test analysis showing the correlation of biotic and abiotic variables with BGC composition, abundance and richness. MC, moisture content; MiCo, microbial composition; PD, microbial phylogenetic diversity.

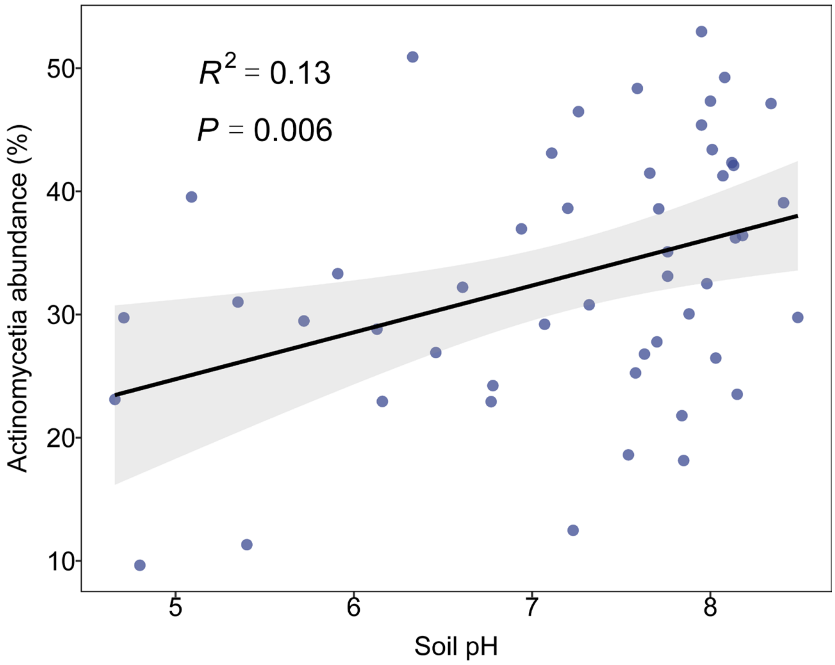

Fig. S8. The correlation between the relative abundance of *Actinomycetia* and soil pH. The relative abundance of *Actinomycetia* was calculated based on mOTU [2] taxonomic profiling of soil metagenomes.

2. Supplementary tables

Table S1. The PERMANOVA test of pairwise vegetation types using pairwiseAdonis package in R.

Pairs	Df	SumsOfSqs	F.Model	R2	p.value	p.adjusted	Sig.
Oilseed vs Rice	1	0.417935	1.56047	0.07977671	0.05	0.05	.
Oilseed vs Wheat	1	0.990828	5.225306	0.2249833	0.001	0.003	*
Oilseed vs Maize	1	0.625703	2.462	0.12032058	0.01	0.012	.
Rice vs Wheat	1	0.897952	3.624466	0.16760951	0.001	0.003	*
Rice vs Maize	1	0.623525	1.996745	0.09985351	0.009	0.012	.
Wheat vs Maize	1	0.595871	2.545745	0.12390618	0.007	0.012	.

Table S2. The significance test of partial RDA-based variance partition analysis.

Group		Df	Variance	F	Pr(>F)	Sig.
	Model	6	0.060576	1.4815	0.001	***
Geographic+Climatic factors	Residual	22	0.149928			
	Model	15	0.15952	1.5605	0.001	***
Microbial composition	Residual	22	0.14993			
	Model	6	0.060528	1.4803	0.003	**
Edaphic properties	Residual	22	0.149928			

Table S3. The network topology of samples grouped by soil moisture or BGC richness.

	Soil moisture			BGC richness		
	Low	Medium	High	Low	Medium	High
Negative edge number	141	156	127	29	56	21
Edge number	3165	6413	5213	3694	2454	944
Negative edge proportion	4.5%	2.4%	2.4%	0.8%	2.3%	2.2%
Node number	1111	1135	1138	1059	1002	762
Clustering coefficient	0.31	0.41	0.42	0.45	0.36	0.31
Average path	5.11	3.97	4.47	6.93	6.02	7.85
Modularity	0.65	0.65	0.69	0.62	0.73	0.83
Graph density	0.05	0.010	0.008	0.007	0.005	0.003
Diameter	21	15	14	26	22	23

Reference

1. Chen, S.; Zhang, C.; Zhang, L., Investigation of the Molecular Landscape of Bacterial Aromatic Polyketides by Global Analysis of Type II Polyketide Synthases. *Angewandte Chemie International Edition* **2022,** *61*, (24), e202202286.

2. Ruscheweyh, H.-J.; Milanese, A.; Paoli, L.; Karcher, N.; Clayssen, Q.; Keller, M. I.; Wirbel, J.; Bork, P.; Mende, D. R.; Zeller, G.; Sunagawa, S., Cultivation-independent genomes greatly expand taxonomic-profiling capabilities of mOTUs across various environments. *Microbiome* **2022,** *10*, (1), 212.
